# The neural basis of vestibular evoked myogenic potentials. The cVEMP is a specific indicator of saccular function

**DOI:** 10.3389/fneur.2025.1644120

**Published:** 2025-08-06

**Authors:** Ian S. Curthoys, Laura Fröhlich, Leigh McGarvie, Leonardo Manzari, Shinichi Iwasaki, Julia Dlugaiczyk, G. Michael Halmagyi

**Affiliations:** ^1^Vestibular Research Laboratory, School of Psychology, The University of Sydney, Sydney, NSW, Australia; ^2^Department of Otorhinolaryngology, Head and Neck Surgery, University Medical Center Bonn, Bonn, Germany; ^3^MSA ENT Academy Center, Cassino, Italy; ^4^Department of Otolaryngology & Head and Neck Surgery, Nagoya City University Graduate School of Medicine, Nagoya, Japan; ^5^Department of Otorhinolaryngology, Head and Neck Surgery & Interdisciplinary Center for Vertigo, Balance and Ocular Motor Disorders, University Hospital Zurich (USZ), University of Zurich (UZH), Zürich, Switzerland; ^6^Neurology Department, Institute of Clinical Neurosciences, Royal Prince Alfred Hospital, Camperdown, NSW, Australia

**Keywords:** VEMP, rat, guinea pig, otolith, utricular, semicircular canal

## Abstract

Vestibular evoked myogenic potentials (VEMPs) are widely used clinical vestibular tests and their interpretation is derived from the original proposal by Colebatch that the cVEMP is due to saccular activation by air conducted sound (ACS). This was based on previous extensive evidence that sounds selectively activate saccular afferent neurons and not semicircular canal neurons at clinical testing conditions. We revisit that earlier data and the results since. Despite that evidence, a recent partial review of the neural basis of cVEMPs has raised the possibility that canal afferents may be activated at clinical test frequencies and intensities and contribute to VEMPs, which would weaken their clinical specificity. This possibility is largely based on evidence that ACS clicks activate canal afferents in the rat – but not in the guinea pig. We show that result from the rat study is due to the very high sound pressure levels used – intensities which were far higher than those in the guinea pig study. When ACS stimuli of comparable intensity are used for both species at comparable clinically effective frequencies and intensities (~110 dB SPL), otolithic neurons are activated in both species but canal activation by ACS clicks is negligible (and so most probably in humans also). Furthermore, the evidence from lesion and electrical stimulation studies and human clinical data complements the neural evidence and confirms the cVEMP to ACS is a specific test of saccular function. Audibility curves show rats have very high thresholds for frequencies most commonly used for human VEMP testing (500–1000 Hz). That result questions the applicability of results of sound evoked vestibular responses from rat for understanding human vestibular function. There is a range of stimulus intensities and frequencies appropriate for measuring VEMPs. Using stimulus values within this range, neural evidence from rats and guinea pigs shows minimal contribution of canal afferents, meaning that VEMPs are specific tests of otolith function and that any contribution from semicircular canals to human cVEMPs (tones at 500 Hz, clicks at 100 dB nHL) is negligible. Using stimulus values outside that range will stimulate semicircular canal afferents as well as otolith afferents, thus compromising the otolithic specificity of VEMPs.

## Introduction

The traditional ways of clinical testing of vestibular function using angular and linear acceleration have now been complemented by methods which measure vestibular evoked myogenic potentials (VEMPs) in response to air conducted sound (ACS) or bone conducted vibration (BCV). For recent reviews see (1–3). In what was a remarkable development Colebatch et al. showed that ACS click stimulation or BCV from light taps with a tendon hammer caused small myogenic potentials recorded by surface electrodes on the tensed sternocleidomastoid muscle (SCM) (4, 5). This response is now known as the cervical VEMP (cVEMP). Prior histopathological and neural data from Aran, Didier and Cazals (reviewed below) together with data from human clinical neurophysiology (6, 7) led Colebatch to propose that cVEMPs were probably caused by saccular receptors and afferents. This was a remarkable discovery because Colebatch had suddenly introduced a clinical test of a part of the vestibular labyrinth whose function clinicians had not been able to assess before. cVEMPs were rapidly adopted and are now part of routine clinical vestibular testing. Later it was shown cVEMPs are more effectively elicited by brief (7 ms) 500 Hz tone bursts of either ACS or BCV than clicks (8, 9). About 10 years after the introduction of cVEMPs, Rosengren et al. reported another VEMP recorded by surface electrodes close to the inferior oblique (IO) eye muscles in response to ACS and BCV termed the ocular vestibular evoked myogenic potential – the oVEMP – and suggested that its origin may be from utricular macula (10).

**Figure 1:**
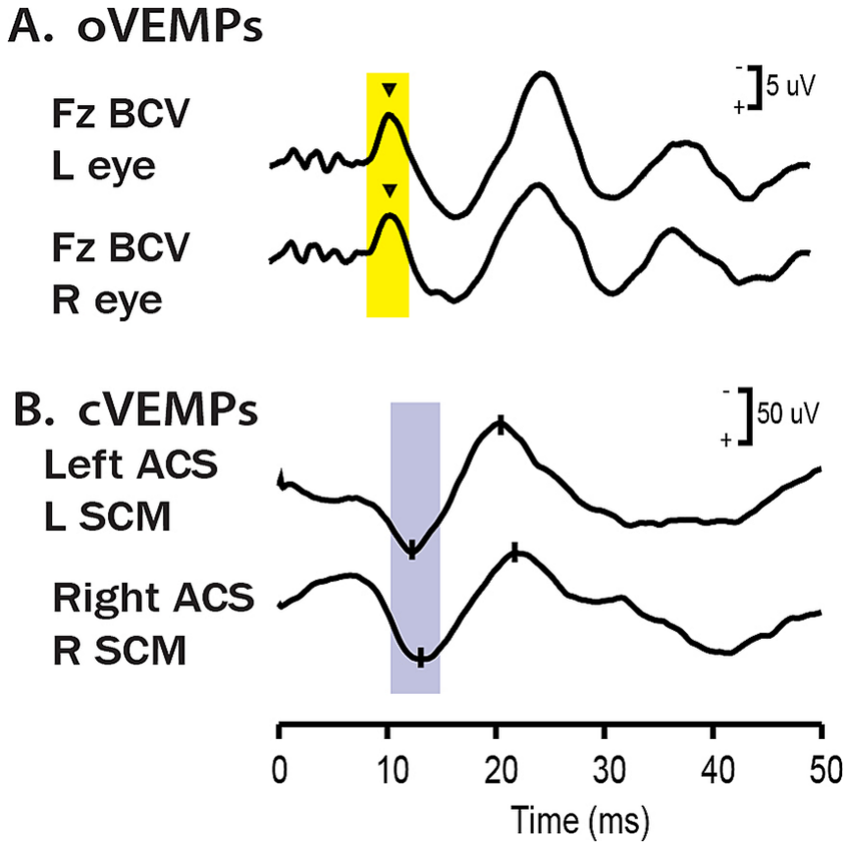
Examples of averaged EMG responses, showing **(A)** oVEMP response to BCV at Fz and **(B)** cVEMP responses to monaural ACS stimulation for a healthy subject. The yellow and blue bands mark the time of healthy oVEMP n10 and cVEMP p13 responses, respectively. Note the different voltage scales for oVEMP vs. cVEMP. The healthy subject displays oVEMP n10 responses of similar magnitude beneath both eyes and symmetrical p13–n23 cVEMP responses. Reproduced with permission from Wiley from (21).

These two types of VEMPs are distinctly different. The cVEMP in response to ACS clicks or BCV stimuli is a short latency positive (inhibitory) myogenic potential recorded on the tensed sternocleidomastoid (SCM) muscle (Figure 1). The ocular VEMP (oVEMP) is a small negative (excitatory) myogenic potential recorded by surface electrodes beneath the eyes as the person looks up. Both VEMPs are extremely fast: in humans the latency from stimulus onset to the onset of the diagnostically important p13 potential of the cVEMP is only about 8 ms (4). The latency from the onset of the stimulus to the foot of the diagnostically important negative n10 potential of the oVEMP is about 6–7 ms (11). In dealing with the neural origin of VEMPs this extremely fast response must be explained.

Are these VEMPs really measures of vestibular function? Colebatch et al. answered that conclusively by showing that patients without hearing exhibited cVEMPs and conversely patients after selective vestibular nerve section and who could hear the stimuli, had no cVEMPs (1). So cVEMPs are a vestibular test and have proved to be a valuable test in the clinical evaluation of patients with balance symptoms, as well as evaluating otolithic function before and after surgical procedures such as cochlear implantion (12).

Vestibular evoked myogenic potentials are of particular clinical value since they test the function of receptors at the striolae of the otolithic maculae which are such key indicators of vestibular function. Type I receptors at the striola are the most metabolically vulnerable of all otolithic receptors – they are the first to show signs of injury after vestibulotoxic antibiotics such as gentamicin (13, 14). The reduction in VEMP response or VOR gain in the video head impulse test (vHIT) in affected ears is of particular value in titrating the dose of intratympanic gentamicin used to treat patients with intractable vertigo (due to, e.g., Menière’s Disease) by producing modest unilateral vestibular loss in order to alleviate vertigo (15–18).

**Figure 2:**
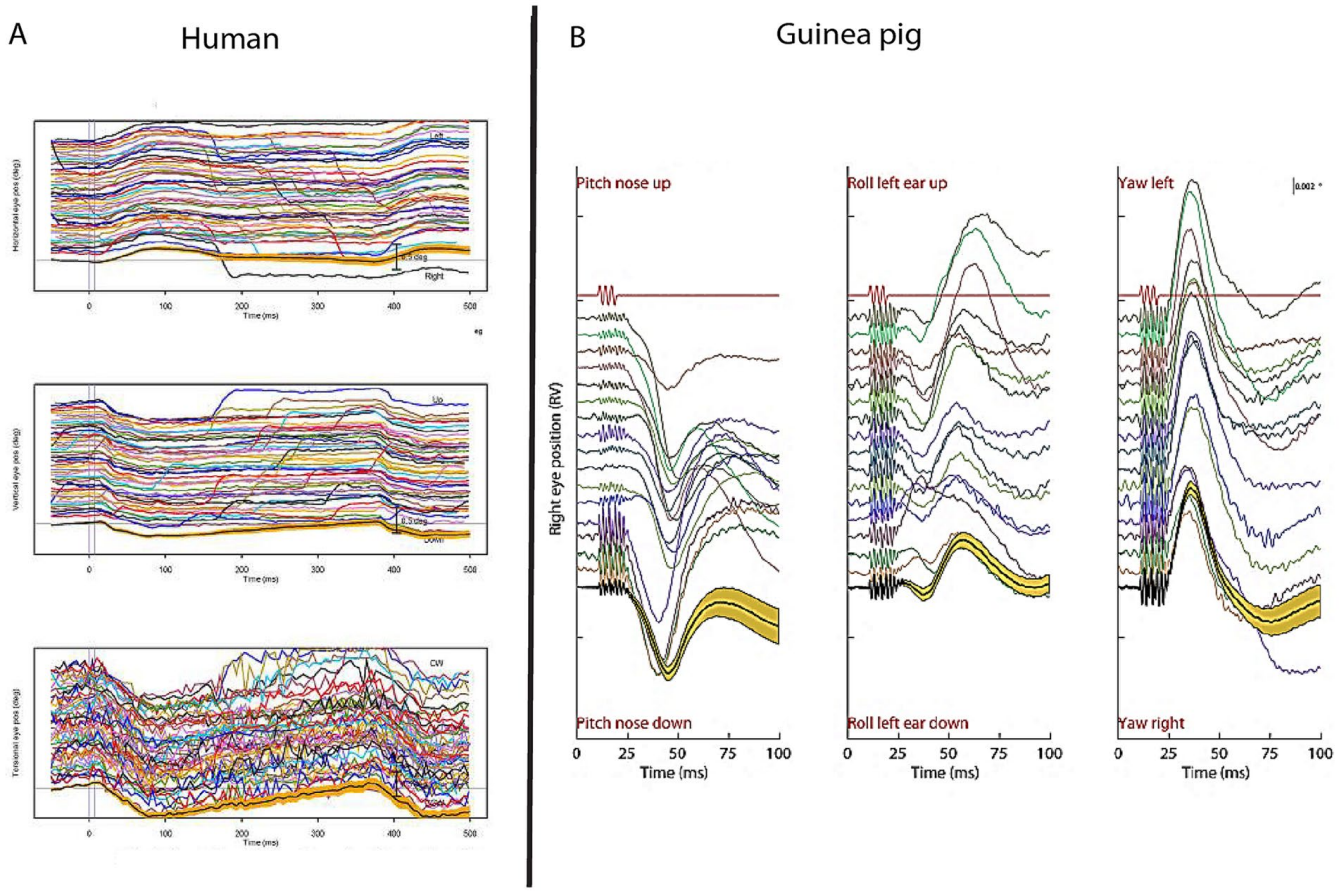
To show the fast and highly repeatable eye movement responses of humans and guinea pigs to 500 Hz BCV stimulation. **(A)** 3D eye position records from the right eye in one human subject during successive presentations of right mastoid stimulation by 7 ms tone bursts of 500 Hz BCV by a B71 bone oscillator. The bottom record in each panel shows the mean values and 95% confidence intervals of the individual responses above. Horizontal vertical and torsional eye movement components (from top to bottom) were measured by pupil and iris tracking using high resolution video acquired at 180 Hz. At a short latency in response to the 500 Hz tone burst (vertical lines) the eye moves horizontally and torsionally away from the side of stimulation. The vertical component is downwards. Replotted from data published in (21). **(B)** To show that brief 500 Hz BCV pulses elicit comparable systematic 3D eye movement responses at short latencies in a guinea pig. Each line shows eye position versus time in response to ten consecutive 500 Hz BCV tone bursts from an alert guinea pig using scleral search coils to record 3D eye movement. The first line in red shows the command voltage for the stimulus, and it defines stimulus onset and duration. The panels show the vertical, torsional and horizontal eye position components (from left to right) of the response on each trial to the tone burst. The final line in each panel shows the mean and 95% confidence interval for the responses. Replotted from data published in (25).

Afferents from utricular and saccular macula have many complex overlapping but differential neural projections (19, 20). The utricular macula has an indirect excitatory projection mainly to the contralateral inferior oblique (IO) eye muscle whereas the saccular macula has a disynaptic inhibitory projection mainly to the ipsilateral sternocleidomastoid (SCM) muscle (see (21) and further discussion below). If this indirect projection from the utricular macula is correct then there should be small, fast eye movements in response to each repeated mastoid vibration stimulus which, on this account, would stimulate utricular receptors and afferents. And there are (see Figure 2A). This was shown by high resolution 3D video recordings of human eye movements in response to brief repeated 500 Hz mastoid stimuli (Figure 2A) which caused small but highly repeatable stimulus locked horizontal, vertical and torsional eye movements (22–24) analogous to the eye movements in cats reported by Suzuki to electrical stimulation of utricular nerve (20). Guinea pigs show similar eye movement responses to similar brief vibration stimulation [Figure 2B; (25)].

Given that VEMPs are vestibular in origin, which vestibular sensory regions are activated by sound and vibration? That requires evidence from single neuron recordings of primary vestibular afferents. As noted above, some early neural evidence was available to Colebatch et al. and much more has appeared since, as we discuss below.

### Didier and Cazals

Colebatch proposed that the cVEMP was due to saccular activation (4). This proposal was based on the wealth of prior behavioral, electrophysiological and histopathological evidence in animals, recording sound evoked responses on the saccular nerve after treatment by cochleototoxic antibiotics (amikacin) which killed cochlear receptors, thus directly implicating the saccule in sound evoked responses and specifically ruling out the potential role of the semicircular canals: McGee and Olszewski - histopathology and behavior (26); Didier and Cazals - histopathology and electrophysiology (27). This wealth of evidence was used by Bickford et al. (6) and Townsend and Cody (7) to implicate the saccular macula as being responsible for the sound evoked response measured at the Inion in humans. In light of this wealth of evidence implicating the saccule in sound evoked responses, Colebatch et al. proposed that saccular receptors and afferents were responsible for cVEMPs (4).

Probably the strongest evidence for the role of the saccule came from the electrophysiological evidence from the Bordeaux group over a period of 10 years of research. These researchers reported that in response to ACS click stimuli in guinea pigs, compound action potentials to ACS clicks were recorded on the saccular nerve after total ablation of cochlear receptors by various ototoxic antibiotics including amikacin and sisomycin (27–32). They showed conclusively that amikacin killed all cochlear receptors, but they could still record a short latency sound-evoked neural response on the saccular nerve. This sound-evoked neural response was clearly not cochlear, and they argued it was due to saccular activation. The Bordeaux group ruled out the semicircular canals as the source of this acoustic response because canal damage in these treated animals did not affect what they described as this “peculiar” acoustic response: “But in cases of total cochlear and drastic ampullar and utricular destruction together with an almost undamaged saccular sensory epithelium the same peculiar acoustic responses could be observed.” Cazals et al. further stated: “These results support the hypothesis of a functional acoustic reception by the saccule in a mammal” (31), p. 211. Years later Murofushi and Matsuzaki confirmed that a VEMP-like response could be recorded on neck muscles in guinea pigs after treatment with the same dose of the cochleotoxic amikacin that the Bordeaux group had used (33).

In an amazingly foresightful conclusion, they stated: “these data favor the hypothesis of a hearing function of the saccule in a mammal possibly involving type 1 cells and electrical synapses.” (31) p. 216. [Recent evidence shows that membrane potential-dependent electrical transmission at the calyx afferent ending enveloping type I receptors is responsible for the extremely fast transmission across that synapse (34–36)]. McGee et al. and Townsend et al. earlier had also specifically excluded semicircular canals as having a role (7, 26).

It is surprising and puzzling that this very extensive and directly relevant evidence of the saccular origin of sound evoked vestibular responses by the Bordeaux group was not referenced in a recent review purportedly of the neural origin of cVEMPs (37). As we show below, even more evidence has further confirmed the saccular specificity of cVEMPs to ACS or BCV.

Translating data from animal physiological experiments to human clinical vestibular function requires careful consideration of many aspects (38). This review integrates that old and new knowledge and explains a number of errors, omissions and misconceptions in this field and explains some of the apparent disagreements about ACS click activation of primary vestibular afferents. As well as the neural evidence there have been a number of results from experimental and clinical testing, which directly support the saccular origin of cVEMPs.

## Neurophysiological evidence from different species

**Figure 3:**
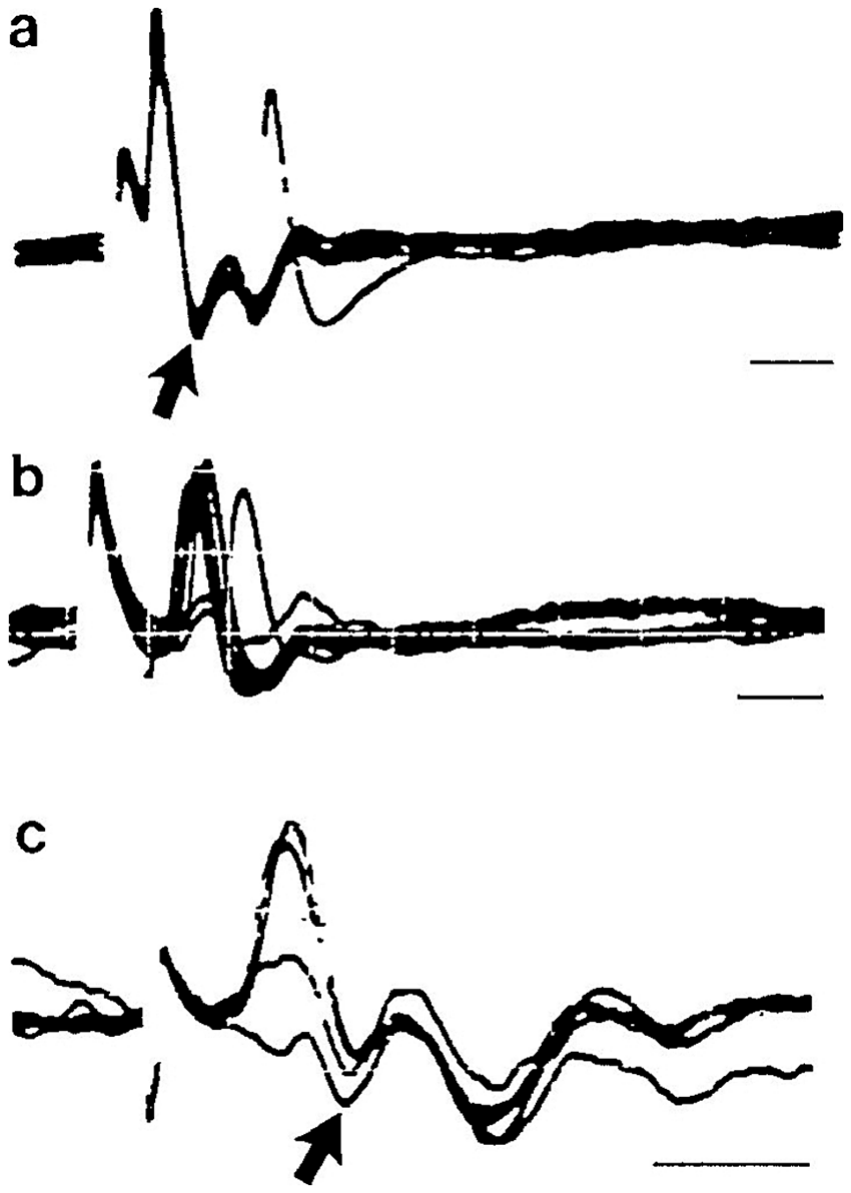
Neural responses to ACS clicks. Time series of responses of 3 guinea pig primary vestibular neurons to ACS clicks, to show the short latency of activation of primary neurons. Each line is the superimposed responses of the neuron to 5 or so stimuli. The latencies of spike activation are 0.5 ms in **(a)** and **(c)** and 1.0 ms in **(b)**. Time scales are 1.0 ms. Reproduced from (41) with permission of Springer Nature.

What is the neural basis of these responses to ACS and BCV which could drive an eye movement response at such a remarkably short latency? Clearly it is the transduction and transmission at the onset of the stimulus which is of paramount important since cVEMPs and oVEMPs are elicited so quickly after the onset of transient stimuli such as brief (2 ms) tone bursts or ACS clicks (39). The rise time of the stimuli is a major determinant of the VEMP amplitude (8, 40). Aspects of the neural response to ACS and BCV which only occur using a maintained stimulus after the initiation of the neural response are irrelevant for the generation of the initial fast response. Murofushi et al. recorded fast activation of irregular neurons by ACS clicks with latencies as short as 0.5 ms (Figure 3) (41). That speed and the significance of stimulus intensity are highlighted throughout this paper.

Different groups studying the vestibular neural response to ACS and BCV have used different species, different stimuli, different criteria of activation to determine whether single primary otolith or canal neurons are activated in response to ACS or BCV. In most of these studies, vestibular neurons are identified by their response to angular or linear accelerations and then ACS or BCV stimuli are presented to test if the neuron responds. If possible, the site of origin of activated neurons is verified by staining the neurons, processing the tissue and so identifying their site of origin, although this is an extremely difficult technique. Since these studies seek to explain the basis of a human response, the stimulus parameters in animal experiments should be selected where possible to be comparable to those used for human clinical testing and the animal species should have comparable sensory characteristics to those of human subjects.

The approach of the Curthoys group has been to record from single neurons in the vestibular nerve in guinea pigs (41–43) whereas the Zhu group has recorded from vestibular neurons in the rat (44, 45). In both species recordings from vestibular afferent neurons usually show most neurons have spontaneous activity at rest, but different neurons have different firing patterns at rest related to their different responses to stimuli. At rest, some neurons generate action potentials at regular intervals (regular neurons), whereas others generate action potentials at irregular intervals (irregular neurons). Regular neurons show excellent responses to maintained linear accelerations but are rarely activated by sound or vibration even at the highest stimulus levels used (44–46). The results of all species which have been studied are that it is otolithic neurons with irregular resting discharge which are activated by sound and vibration at stimulus levels corresponding to those used for human clinical testing (41, 44, 45, 47, 48). Goldberg et al. showed that otolithic neurons with irregular resting discharge originate from amphora shaped type I receptors enveloped by calyx afferent endings at the striola of the utricular macula (49). Recording and tracing studies in guinea pigs confirm that it is irregular afferent neurons from the striola of the utricular and saccular macula which are activated by ACS and BCV (24, 46, 50, 51).

A recent review has raised the issue of the possible activation of semicircular canal neurons by ACS and BCV and whether this canal activation may contribute to standard clinical cVEMPs (37). This is a serious issue since if semicircular canal neurons are activated at clinical test frequencies and intensities, it detracts from the specificity of the cVEMP as a test of saccular function. In the following we discuss this issue and resolve a recent widespread misunderstanding about canal neuron activation by ACS or BCV.

There is a long history showing that vibration is an effective stimulus for otolithic receptors and neurons going back to Ross in 1936 in frogs (52) and later Lowenstein in rays (53) and then Young et al. recording primary vestibular afferents in squirrel monkey (54). Many others since have shown by single neuron recording that otolithic neurons are activated by sound or vibration and in the following we focus on the major reports relevant for the question of the saccular origin of cVEMPs in animals with intact normally encased bony labyrinths: Young in the squirrel monkey, McCue and Guinan in the cat, Murofushi in the guinea pig, Curthoys in the guinea pig and Zhu in the rat.

### Neural evidence from squirrel monkey

Young et al. studied the response of primary vestibular neurons in squirrel monkeys and found single vestibular neurons with irregular resting discharge were activated by ACS and BCV (54). They had two criteria of neural activation. The simplest and most direct indicator was a stimulus-locked audible increase in firing rate – a rate change – in a response to ACS or BCV stimuli. But there was another indicator that the stimuli were affecting the neural response (and so constituting activation). They found stimuli changed the precision of firing of action potentials relative to the stimulus – the phase-locking precision – during long duration 400 ms stimuli without any increase in firing rate. In other words, exactly when the neuron fired during the stimulus shifted, but there was no overall increase in firing rate, so there was no increase in the crucial early synaptic transmission needed to trigger an early myogenic potential and even an eye movement. They compared the two criteria, phase-locking and rate change, in some neurons and found phase-locking threshold was always seen at lower intensity levels compared to the threshold for changes in discharge rate. The rate change thresholds in a sample of 26 responsive neurons were on average 21.5 dB above phase-locking thresholds. They reported that rate change thresholds were so high they could not be determined for most neurons, so Young et al. used phase-locking thresholds as their measure of sensitivity, but as we have stressed, phase-locking is irrelevant for the onset of the response as occurs with VEMPs. Phase-locking does show that the neuron was activated by the stimulus and that is incontrovertible, it is just that it misses the point: phase-locking is not relevant for determining the receptor and neural response at the onset of the stimulus. That early response has to be due to a stimulus-locked action potential triggered by the onset of the stimulus. But since Young was an auditory physiologist and phase-locking was easy to measure, Young et al. used phase-locking precision as their indicator of vestibular activation by ACS and BCV. They realized how counterintuitive this phase-locking measure was–and they said that they doubted the brain could use precision of phase-locking, but for them it was ideal because they could measure it simply. The artificiality of the phase-locking criterion was already recognized by Young et al.: “Most likely, phase-locking at audio frequencies would not, by itself, be recognized as an activation of the vestibular apparatus. If rate changes were required, then the median threshold would exceed 120 dB, an intensity leading to discomfort or pain in humans” (54) p. 358.

The inconsistency of the results of Young et al. using the two criteria is shown by their summary of the results which emphasized that canal afferents had higher phase-locking thresholds than saccular afferents: “In contrast, almost all saccular afferents were affected by sound stimulation; median thresholds at 250 and 350 Hz are between 106 and 110 db. The corresponding values for canal units were at least 120 dB at these frequencies” (54) p. 357. That summary statement is exactly in accord with the main result from later studies reviewed below, that canal afferents have much higher thresholds for sound compared to otolith afferents.

We contend that the widely held belief that the Young et al. study showed canal afferents are more sensitive than otolith afferents should be set aside because it is inconsistent with their textual summary of their own results and because it is based on the use of a criterion of activation which is not relevant for the initiation of the fast neural response to the stimuli within the first 0.5 ms which is crucial for the generation of VEMPs (Figure 3). As they stated themselves, the major result from the Young et al. study is that otolithic neurons are activated at substantially lower intensities compared to canal neurons.

### Neural evidence from guinea pig (ACS)

**Figure 4:**
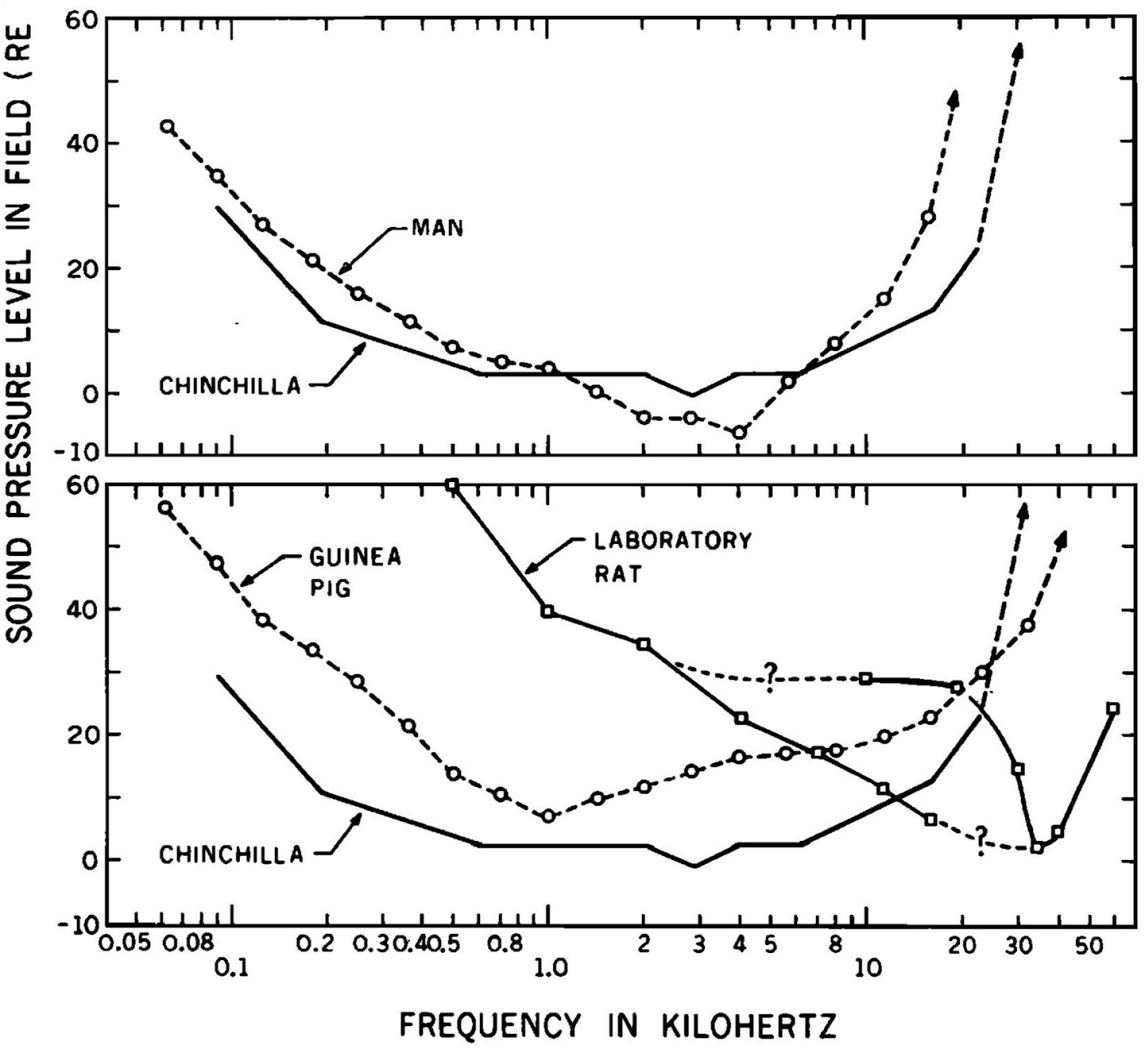
Audibility curves (plots of the sound intensity at threshold in dB SPL for various frequencies) for the chinchilla and human **(upper panel)** and the guinea pig and rat **(lower panel)**. The rat and guinea pig data shows the free field hearing threshold determined by behavioral audiometery for ACS tones of increasing frequency in dB SPL. At 500 Hz the guinea pig threshold is about 15 dB SPL, whereas the rat threshold is about 60 dB SPL – a difference between species of 45 dB. The human threshold at 500 Hz is 8 dB SPL. Reproduced from (55) with permission of the Journal of the Acoustical Society of America.

Murofushi and Curthoys set out to find the cause of cVEMPs to high intensity ACS clicks (41, 42), which was being developed concurrently by Colebatch and Halmagyi in Sydney (4, 5). In anaesthetized guinea pigs we sought neural responses of primary afferents which functioned to generate a reliable, short latency motor response to ACS clicks. The clinical focus of the Murofushi study has been followed since by the Curthoys group. The guinea pig is an ideal animal for such an investigation since guinea pig and human audibility curves are reasonably similar, whereas the rat audibility curve is markedly different (55) (Figure 4). The audibility curve shows the free field threshold for ACS tones of increasing frequency in dB SPL. At 500 Hz the guinea pig threshold is about 15 dB SPL, whereas the rat threshold is about 60 dB SPL – a huge difference between species of 45 dB. The human threshold at 500 Hz is 8 dB SPL. Initially the aim was to test a large number of neurons to identify whether ACS clicks caused short latency activation (increased firing rate) in vestibular neurons, in particular saccular neurons as Colebatch had suggested following Didier and Cazals. The stimulus parameters used were selected to be comparable to the parameters used in human clinical testing, rather than being a wide-ranging archival study of multiple frequencies and intensities.

A crucial decision in this and later studies was the stimulus intensity which corresponded to the high, but not extreme, values being used in the concurrent development of the VEMP for human vestibular testing. If a single high intensity were to be used (such as 130 dB SPL) it would not take account of animals with possible conductive hearing loss and may, as we now know, cause vestibular damage (56). Some guinea pigs, like humans, have conductive hearing loss. This matter was resolved in the following way: Since the guinea pig and human audibility curves have reasonably similar thresholds (Figure 4, dashed lines), we reasoned that the physical intensity of clicks at auditory brainstem response (ABR) threshold could be used as a baseline from which to refer to intensities in order to ensure the guinea pig results were comparable between animals and also comparable to human results. The guinea pig ABR threshold to clicks has been measured at 20–25 dB SPL (25, 57) which is close to the human ABR threshold to clicks [33 dB SPL (58)]. The intensity of ACS clicks used for human clinical VEMP testing is about 70–90 dB above human ABR threshold- i.e. around 100–110 dB SPL (2). So, we used intensities for guinea pigs which were 70–90 dB above guinea pig ABR threshold - i.e. up to about 100–110 dB SPL. This allowed comparison and equilibration of intensities between individual guinea pigs and comparability between guinea pigs and humans. A similar logic was used to justify the use of ABR threshold as a baseline reference for stimulus intensity in later studies on the effect of BCV on vestibular neural responses (48). As we explain below this use of the ABR threshold as a reference or baseline level is crucial in understanding the difference between the results of rats and guinea pigs.

The Murofushi study tested the response of a large number of single neurons from various vestibular sensory regions to identify if they were activated by ACS clicks. What constitutes activation? This is really fundamental since some stimuli may deflect the cilia of vestibular receptors, and so technically activate receptors, but fail to generate stimulus-locked action potentials in primary vestibular afferents that trigger responses in later neurons. Such a stimulus technically activates the receptor, but it is functionally irrelevant for generating the myogenic response we sought to explain, since it does not result in action potentials reaching the myogenic target. We used the simplest most direct indicator of activation, which was a stimulus-locked audible increase in firing rate - a rate change - in response to ACS or BCV stimuli. The results of Murofushi et al. in the guinea pig demonstrated the selectivity of saccular neurons for ACS clicks and the insensitivity of canal neurons for ACS clicks at clinical test intensities.

We demonstrated that otolithic neurons with irregular resting discharge were most likely to be activated by ACS clicks. In the Murofushi et al. study, 102 otolithic neurons were activated by ACS clicks but not one of 300 canal neurons were activated (42). Some activated neurons were stained and traced back to their origin in the saccular macula. We concluded that the saccule responds to acoustic clicks just as the work of the Bordeaux group had indicated and that cVEMPs to ACS clicks test saccular function primarily.

### Neural evidence from cat

McCue and Guinan recorded from irregular afferents in the inferior vestibular nerve of cats and demonstrated that 229 irregular afferent neurons were activated by ACS stimuli (both rate change and phase-locking criteria were used (47, 59)). These neurons had short latencies (around 0.7 ms) as Murofushi et al. had found in guinea pigs, shorter than the latency of cochlear afferents in the cat (which is about 1.0 ms (60)). McCue and Guinan traced two activated neurons to the saccular epithelium. The minimum intensity was high – they reported that there were no responses for stimuli below intensities of 90 dB SPL. No regular afferents were activated. Their results are similar to those of Murofushi et al. 1995 in the guinea pig although the two groups worked independently.

### Neural evidence from chinchilla

In a study of the effect of semicircular canal dehiscence (SCD) on semicircular canal afferent responses, Carey recorded many primary semicircular canal afferents in chinchillas to ACS stimulation and showed activation of canal afferents before SCD by ACS at very high intensities – 135 dB SPL (61). In many of these experiments Carey made an opening (a dehiscence) in the bony wall of the anterior semicircular canal - a procedure referred to as a semicircular canal dehiscence (SCD) – and showed that the SCD resulted in much lower thresholds of activation of canal neurons by sound, as Wit et al. had shown in the pigeon (62) and Mikaelian in the mouse (63). The one irregular canal afferent (of 15 tested) which could be activated with the bony canal intact required the very high stimulus level of 135 dB SPL. After the SCD, irregular canal afferents could be evoked with a much lower average threshold of 96 dB SPL. The insensitivity of canal afferents with intact bony canals to ACS shown by Young and Murofushi et al. was confirmed by the results of (61). Songer and Rosowski (64) showed that the SCD changes the fluid dynamics of the canal and is the cause of the enhanced canal afferent response.

### Neural evidence from guinea pig (BCV)

Halmagyi et al. had shown that BCV from gentle taps by a tendon hammer at the midline of the forehead at the hairline (a location called Fz) is an effective way of generating cVEMPs (48, 65). BCV has the advantages that it can be delivered at much lower subjective intensities, is not unpleasant, and is effective in that the abrupt change in linear acceleration (jerk) as the hammer hits the skull delivers clear results unencumbered by the major problem of ACS stimulation – impairment of sound transmission through the middle and/or inner ear so that the sound energy is not adequately transmitted towards the vestibular system. But the same question arose – is BCV a specific otolithic stimulus? So, the Curthoys group sought evidence that BCV was a selective otolithic stimulus (48).

In the 2006 study a large number of primary vestibular afferents in guinea pigs were recorded, testing the response of every one to brief (7 ms) 500 Hz tone burst stimuli - the same frequency Welgampola et al. had used to test cVEMPs to BCV in human patients (48, 66). Activation was identified by listening for a change in firing rate, locked to the search stimulus, which was delivered by a clinical bone oscillator (Radioear B71 cemented to the guinea pig skull) at intensities up to 90 dB above the animal’s ABR threshold. For a neuron to be classified as activated there had to be an audibly detectable increase in firing rate to this search stimulus, in other words, a clear, reliable, stimulus-locked increase in firing rate, in order to be called activation, strong enough to trigger an eye movement on every single 7 ms vibration stimulation, as repeated 7 ms BCV stimulation of the human mastoid does (and also in guinea pigs, see Figure 2). These records show that a short latency eye movement is produced on every single stimulus presentation both for humans and guinea pigs, so activation at the very onset of the stimulus must be effective in every single case in generating this response.

**Figure 5:**
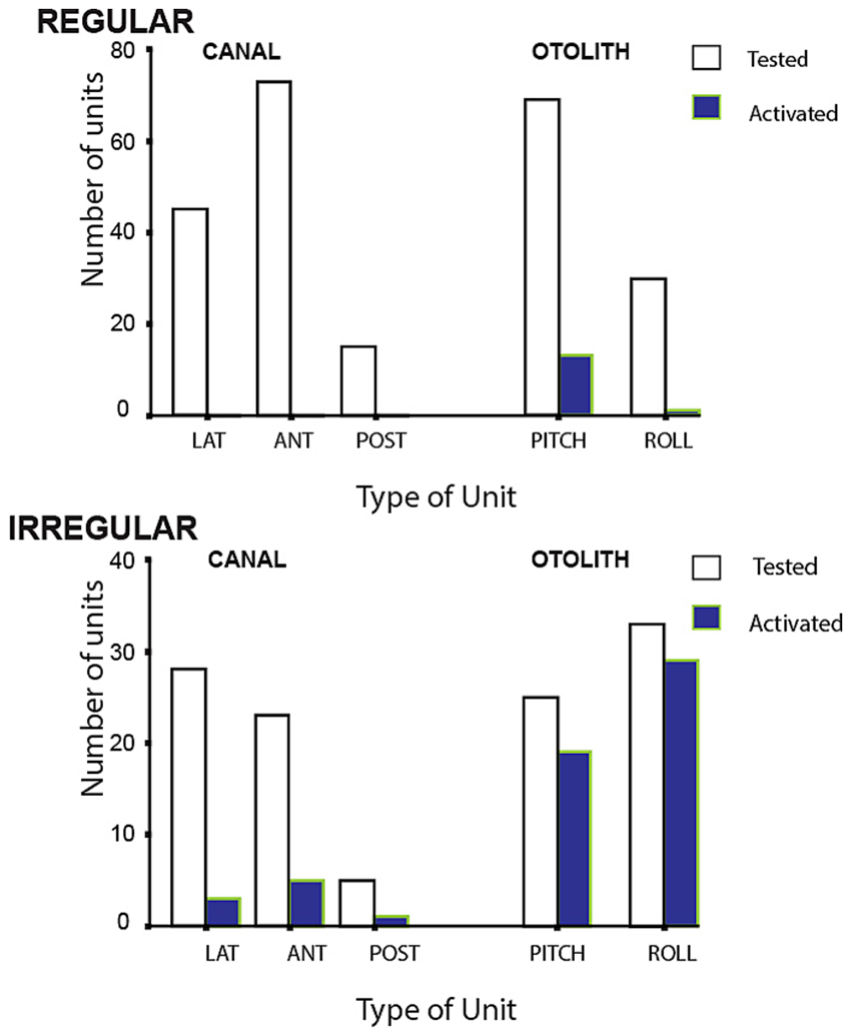
Numbers of regular and irregular primary vestibular afferent neurons tested (open bars) and activated (closed bars) by 500 Hz bone conducted vibration at 80 dB above guinea pig ABR threshold. Lat, horizontal canal; Ant, anterior canal; Post, posterior canal. The otolith neurons are divided into Pitch static and Roll static. The results show that only a small proportion of semicircular canal neurons were activated at this intensity, in contrast a large proportion of otolithic irregular neurons tested were activated. Otolith and canal regular neurons were rarely activated. Reproduced from (48) with permission of Springer Nature.

The results of BCV stimulation showed 82.8% of irregular otolithic afferents tested, but only 4.7% of irregular canal neurons tested were activated by the 500 Hz stimulus (see Figure 5). In later studies using lower frequencies, discussed below, we (67) demonstrated that in animals with bony labyrinth intact as normal (i.e., no semicircular canal dehiscence), irregular semicircular canal afferents as well as otolithic afferents are activated by low frequency vibration (<200 Hz), but not by 500 Hz BCV.

Neurons with irregular resting discharge showed a clear increase in firing rate to 500 Hz BCV at low stimulus levels and staining and tracing showed that these irregular afferents originated from the striola of the utricular macula as Goldberg et al. had reported (49), but also from the striola of the saccular macula (48). Neurons with regular spontaneous activity showed no detectable increase in firing rate to 500 Hz BCV up to the maximum we used (2 g). Regular neurons show excellent responses to maintained or low frequency linear accelerations however they did not respond to ACS or BCV at the clinically effective stimulus levels we used. In similar fashion, regular canal neurons were rarely activated by 500 Hz BCV (Figure 5).

### Neural evidence from rat (ACS)

In 2011 Zhu et al. published a paper showing that ACS clicks at a single high intensity (130 dB SPL) activated both irregular otolith and canal neurons in rats (44, 45). This appeared to be an explicit contradiction of the results of Murofushi et al. (41, 42) (and also the later Curthoys et al. study with BCV stimuli) (48) both of whom had found little activation in canal afferents. Zhu et al. used a technique which is likely to capture even the smallest possibility that a neuron was activated by sound: a cumulative probability of just 10% of a spike across 150 successive ACS click presentations at 130 dB SPL. Every neuron encountered was tested with this high intensity barrage in order to identify if it was activated. This entails that for every animal there was a significant level of exposure to high intensity clicks (at 130 dB SPL) since many neurons were tested in each animal. Exposure to high intensity ACS stimuli has been shown to affect vestibular function (56, 68). The criterion used by the Curthoys group was much more conservative: there had to be a clear stimulus-locked increase in firing during each search stimulus presentation and that occurred at low BCV intensities a as a later study of thresholds showed (50).

The results of the Zhu et al. (45) study implied that ACS clicks were not specific otolithic stimuli as had been reported by Murofushi et al. [and later for BCV stimuli Curthoys al. (48)] since ACS clicks in their experiment activated both otolithic and canal afferents. Such a result brought into question the specificity of the cVEMP in response to ACS clicks as a test of saccular function and the results of Zhu et al. (45) have been used as a basis for questioning the specificity of cVEMP testing by ACS (37). This apparent contradiction between the results of Murofushi and Zhu has continued for years but more extensive results published later by the Zhu group in 2014 show the probable reason for this apparent disagreement (44).

In the original Zhu 2011 paper only one high intensity test stimulus was used −130 dB SPL which was called 80 dB SL (45). The term SL means that the intensity of every click stimulus was 80 dB above an arbitrary reference level, which was the click intensity at rat ABR threshold. As we noted above referring stimulus intensity to ABR threshold had been used in guinea pigs (41) and Zhu et al. (45) followed that idea and even the value used (80 dB). So, the stimulus intensity for both rat and guinea pigs was 80 dB above ABR threshold. However, there is a major difference between the physical sound intensity at rat and guinea pig ABR thresholds. The physical click intensity at ABR threshold for the rat is surprisingly high - about 50 dB SPL (56), whereas for the guinea pig, the intensity at ABR threshold is much lower around 20 dB SPL (25, 57). The high ABR threshold for the rat means that the click intensity used in the Zhu et al. (45) study at 80 dB above the 50 dB SPL threshold was very high – i.e. 130 dB SPL, whereas the click intensity for the guinea pigs in the Murofushi was much lower (80 dB above 20 dB - 100 dB SPL). So, there was a substantial difference in the stimulus click intensity of about 30 dB between the two species, although both were at 80 dB above ABR threshold. 130 dB SPL is close to the highest intensity recommended for human clinical vestibular testing (2). In summary we suggest that the probable reasons that Zhu et al. (45) found such extensive canal activation in the rat compared to guinea pig, is the criterion for neural activation and that the stimuli used for the rat in the 2011 study were a much higher physical intensity than the stimuli used for the guinea pig.

**Figure 6:**
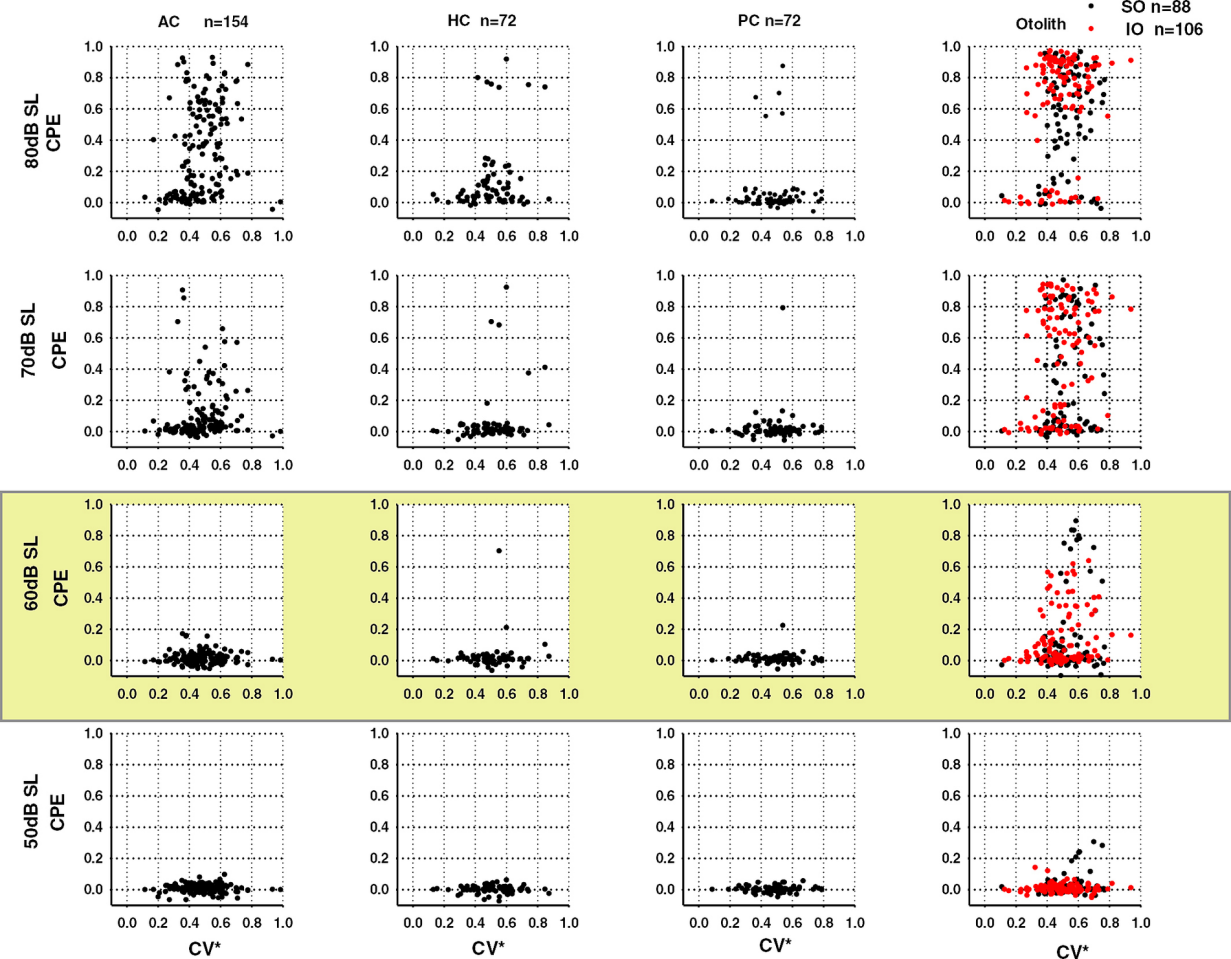
Plots of neural activation (CPE - cumulative probability of evoking a spike) of rat canal and otolith neurons in response to ACS clicks as a function of regularity of resting discharge (CV*). The rows show the results for 4 levels of ACS click intensity: 50, 60, 70, 80 dB above the rat ABR threshold (SL). In every graph each dot shows the probability of firing of an individual neuron to 150 click presentations. The four columns show the results for neurons of the anterior (AC), horizontal (HC), and posterior canals (PC) and otoliths. At high intensity (top row at 80 dB SL) many anterior canal as well as otolithic neurons show a high probability of activation, corresponding to the results at that stimulus intensity reported earlier by (45). The highlighted row (60 dB SL) shows the results at the ACS stimulus intensity comparable to the stimulus intensity used in the Murofushi et al. study of guinea pig afferent neurons (41, 42). At this stimulus level otolithic neurons are activated (column 4), but few canal neurons are (columns 1–3). That result is comparable to the result reported by Murofushi et al. (41) in guinea pigs. Reproduced from (44) with permission of Springer Nature.

That suggestion is confirmed by the later published data from the Zhu group (44) where they reported the results from rat ACS click experiments using similar testing paradigms but using stimuli of lower intensities (comparable to the intensities used in the Murofushi et al. guinea pig study). They found that at lower intensities (e.g., 60 dB SL for the rat – i.e. about 110 dB SPL – which was the upper intensity used in the Murofushi study) there was otolith activation but very little canal activation, corresponding to the guinea pig results of Murofushi et al. (see Figure 6, highlighted row). In sum the probable reason for the apparent contradiction about canal activation by ACS between guinea pig and rat is due to the use of different stimulus intensities in the two species. When stimuli of comparable lower physical intensity are used, the results are comparable in that they both show otolith activation by ACS clicks but little canal activation. These lower intensities correspond to the stimulus levels used for clinical testing, so in the usual cVEMP human clinical test there is likely little neural activation of semicircular canal afferents. Indeed Zhu et al. recognized the problem with high intensities and recommended using lower intensities for clinical testing to minimize the chance of spread to canal afferents “Clicks at or below 60 dB SL activated only otolith organ afferents.” (44) p. 73. Zhu et al. conclude that sound activation of the vestibular end organs other than the saccule should not be ruled out when designing and interpreting clinical VEMP tests.

Recently the Zhu group have published evidence using their characteristic testing paradigm reporting the effects of ACS stimulus frequency on primary vestibular neural activation. Huang et al. used a range of frequencies to explore activation of vestibular neurons by ACS in rats (69). Curthoys et al. have published comparable data about stimulus frequency of BCV stimuli on firing rate threshold of primary otolithic afferents of guinea pig (50, 51, 70). In the Huang rat paper, a new aspect of the difference between rat and guinea pigs (and also humans) was revealed. Huang found that in irregular afferents there is almost no activation of vestibular neurons (otolith or canal) by ACS stimuli at frequencies of 350 Hz or lower (69). In contrast in the guinea pig, otolithic afferents are activated by 500 Hz ACS and have low threshold at 500 Hz (50). These results are to be expected from the audibility data of rats (Figure 4) - rats have an extremely high hearing threshold (about 60 dB SPL) for 500 Hz which is the most common frequency used in human clinical vestibular testing whereas the guinea pig threshold at 500 Hz is about 15 dB SPL (Figure 4) and comparable to human ACS threshold at this frequency. In summary, in the Huang et al. data at 350 Hz, few otolith or canal neurons in the rat are activated by the stimulus frequency which is close to the most commonly used in human clinical VEMP studies (500 Hz) (2). The results in rats to ACS stimuli are so far removed from human results that we conclude that the rat is not a good model for understanding human clinical sound-evoked vestibular responses.

## Relating neural data to VEMPs

The early neural and clinical evidence was assembled in a review in which the following hypothesis was proposed: that cVEMPs are mainly due to saccular function and oVEMPs are mainly due to utricular function (71). That hypothesis was based on evidence that single primary otolithic afferents from both utricular and saccular maculae were activated by both ACS and BCV [Figure 4 of (71), and later (51)]. In other words, it is possible to probe saccular and utricular function separately because of their differential neural projections and so their different myogenic responses to stimulation, either ACS or BCV. The evidence from single neuron recording shows very clearly that ACS or BCV activate afferent neurons from both otolithic sense organs (51, 71). So, the response to ACS indicates saccular or utricular function depending on which *response*, oVEMP or cVEMP, is being measured. The evidence for differential projections of utricular and saccular maculae is overwhelming (19): saccular neurons have a strong projection to neck muscles (72, 73) but a weak projection to the oculomotor system (74, 75) and conversely that utricular afferents have a strong projection to eye muscles (20, 76, 77). Uchino and Kushiro summarized many years of research on otolith-ocular and otolith-spinal projections from Uchino’s group by stating: “Consequently, the neural connections in the sacculo-ocular system are relatively weak compared to the neural connections in the utriculo-ocular and sacculo-collic systems.” [Uchino and Kushiro (19), p. 321]. Uchino also emphasized the greater number of saccular afferents to SCM compared to utricular afferents to SCM. [A recent partial review (37) overlooked the above evidence and the unambiguous statement from Uchino who spent 15 years investigating utricular and saccular projections].

The independence of oVEMPs and cVEMPs was confirmed by the fact that some patients have present oVEMPs but reduced or absent cVEMPs and other patients show exactly the converse (78). Also, patients with superior vestibular neuritis have reduced or absent horizontal canal function and reduced or absent contralateral oVEMPs, but no detectable decrease of ipsilateral cVEMPs (79, 80). Based on the neurophysiological and anatomical evidence outlined above, cVEMPs are employed as a test of mainly saccular function and oVEMPs mainly test utricular function (2, 81–84).

## Discussion

### Canal contributions to VEMPs—after SCD

**Figure 7:**
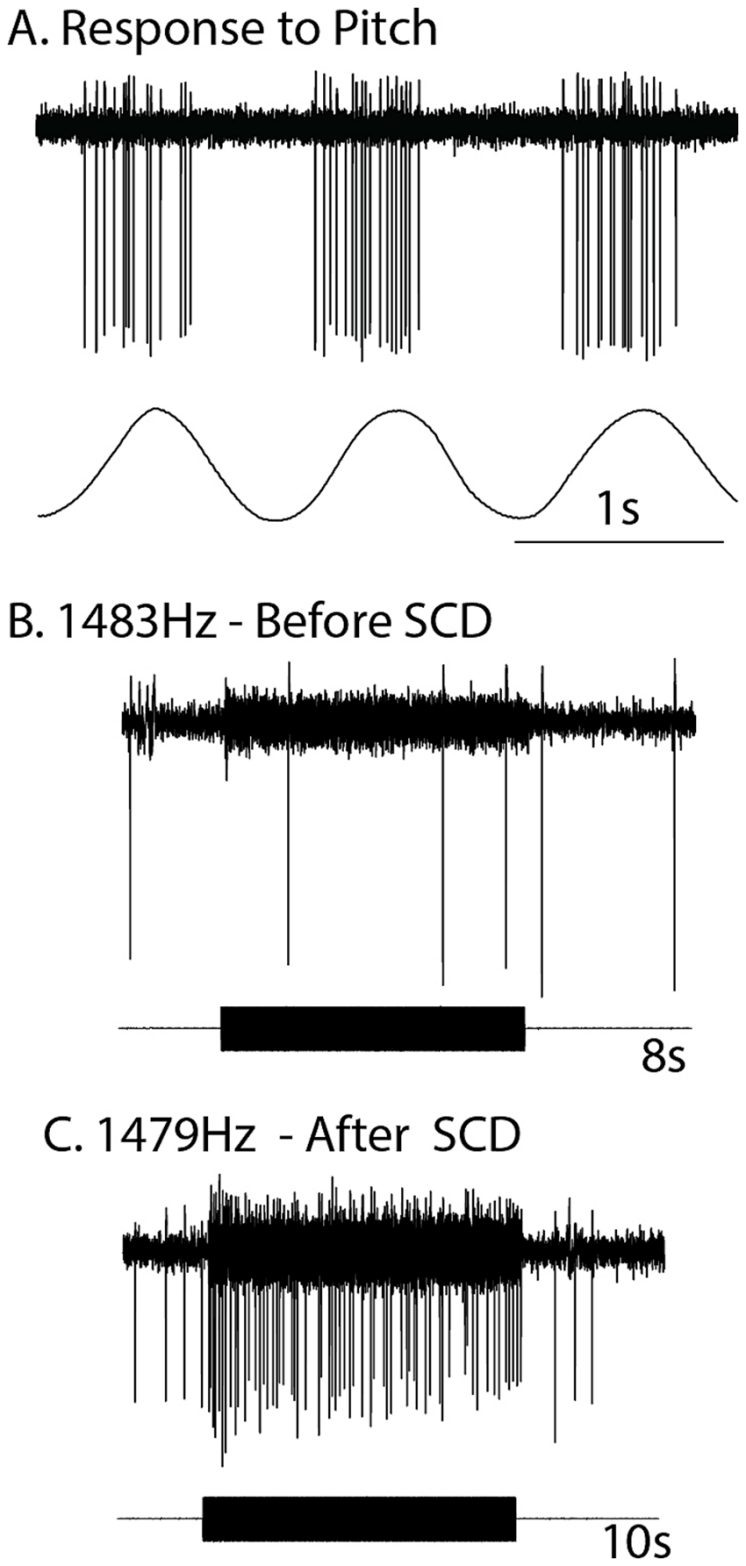
The response of an irregular primary anterior canal neuron to high-frequency ACS before and after a small (0.1 mm diameter) dehiscence (SCD) in the bony wall of the anterior canal. **(A)** The response of the neuron to pitch angular acceleration (lower record) shows that the neuron is an anterior canal afferent. **(B)** Before SCD an 8 s burst of 1,483 Hz ACS has no effect on the neural response. **(C)** After the SCD a 10 s burst of 1,479 ACS causes strong stimulus-locked activation in this anterior canal Hz neuron. The lower records in **(B,C)** show the stimulus command voltage. After resealing the SCD, the response to ACS stimulation disappears (not shown). Reproduced from (85) with permission of Springer Nature. This tiny dehiscence completely changes labyrinth operation.

**Figure 8:**
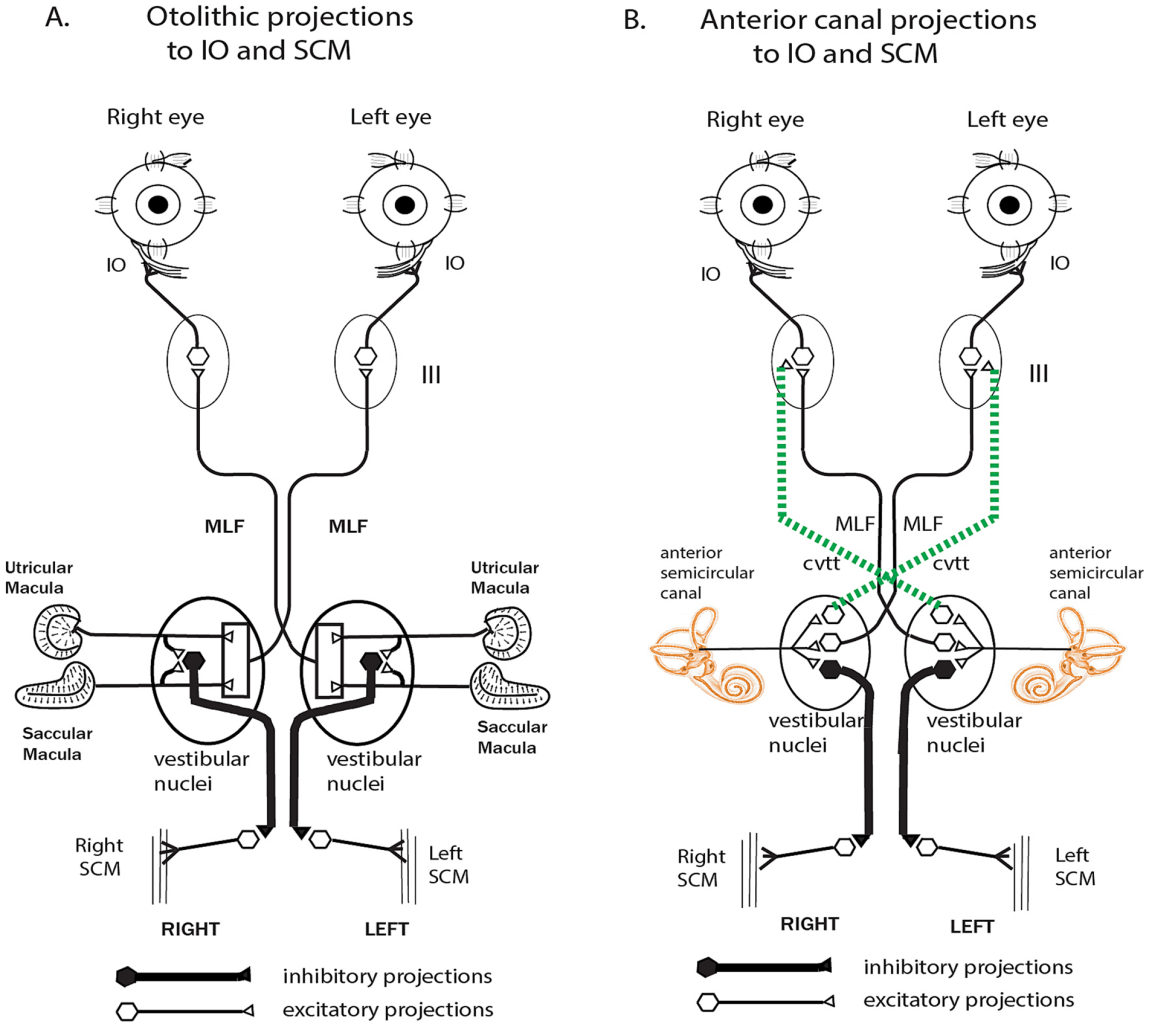
**(A)** Schematic diagram of the projections from the otoliths to the inferior oblique (IO) and sternocleidomastoid muscle (SCM) which likely underlie the oVEMP and cVEMP, respectively. Both schematics are based on the results of (20) and Uchino’s group [summarized in (19)]. **(B)** Projections of anterior semicircular canal neurons to the IO and SCM. In healthy subjects with intact bony labyrinths, stimulation by ACS or BCV will activate otolithic projections only, resulting in oVEMP in IO and cVEMP in SCM. After a dehiscence of the bony wall of a semicircular canal, the same otolithic projections are activated [with even lower thresholds (86)], but since canal neurons are also activated by ACS or BCV after an SCD (as explained in the text) the anterior semicircular canal afferents **(B)** are activated and so will also contribute to the oVEMP and cVEMP. It appears that it is this combination of otolithic and canal afferent activation after SCD which results in the enhanced oVEMP and cVEMP responses after SCD including the oVEMP to 4,000 Hz. Reproduced from (85) with permission of Springer Nature.

While a main conclusion of this paper is that saccular function is tested by the cVEMP, perhaps canal neurons may also contribute to the cVEMP? Can semicircular canal neurons be activated at all by sound and vibration? The answer is yes - depending on the stimulus and bony canal characteristics. That capability is shown by the evidence from responses of canal neurons to high intensity click stimuli (45), and also after an SCD, as we have noted above. Carey et al. (2004) had demonstrated the enhanced canal neural responses in chinchilla after an SCD (61). The definitive data, recording from the same neuron before and after making the dehiscence and also after resealing the dehiscence, provided evidence that individual semicircular canal afferents while unresponsive to ACS tones prior to the SCD, are capable of responding to ACS and BCV up to high frequencies after the SCD (85, 86). Most canal neurons are not activated by 100 dB SPL 500 Hz ACS (or BCV) before dehiscence, after an SCD the same canal neurons which had been unresponsive prior to the SCD, can be activated even by surprisingly high frequencies - frequencies greater than 1,000 Hz. That was clearly shown by recording from single semicircular canal neurons in guinea pigs and making an artificial dehiscence in the bony canal wall whilst recording from the one neuron - testing the one neuron before and after that dehiscence and then sealing the dehiscence and testing the neuron again. In the example shown, the clear effect of a dehiscence as small as 0.1 mm was that before SCD there was no activation to ACS at 500 Hz or to other high frequencies (e.g., 1,483 Hz), whereas after the SCD there was activation of this same neuron at the high frequency (1,479 Hz) (Figure 7). After resealing there was no response to 500 Hz or high frequency stimuli. Testing with angular accelerations after the SCD showed that the neuron had clear responses to pitch angular acceleration, so the membranous canal had not been damaged. That is clear evidence that the tiny dehiscence had changed the mode of labyrinth operation completely by increasing the fluid displacement caused by the stimuli (87, 88). This evidence shows that canal neurons can, in these unusual conditions, respond to vibration and sound. Recording before and after an SCD was repeated in around 70 neurons with similar results (86). The sensitivity of otolith neurons to ACS and BCV also increased after an SCD (86). These results explain the clinical evidence of enhanced oVEMP and cVEMP responses in human patients with an SCD – after an SCD the enhanced oVEMP is the result of the combined activation of otolithic and also the now activated canal neurons (Figure 8), so the enhanced VEMP response is reflecting the summed activation of otoliths and canals (85). The neural activation of canal neurons after an SCD by very high frequencies explains the clinical identification of SCD in patients by the presence of an oVEMP response to 4,000 Hz (89, 90). Interestingly, such “microSCDs” may explain the oVEMP to 4,000 Hz in patients in whom CT does not detect an SCD, because the tiny size of the SCD is below the resolution limit of most CT systems.

### Canal contributions to VEMPs–low frequency vibration

**Figure 9:**
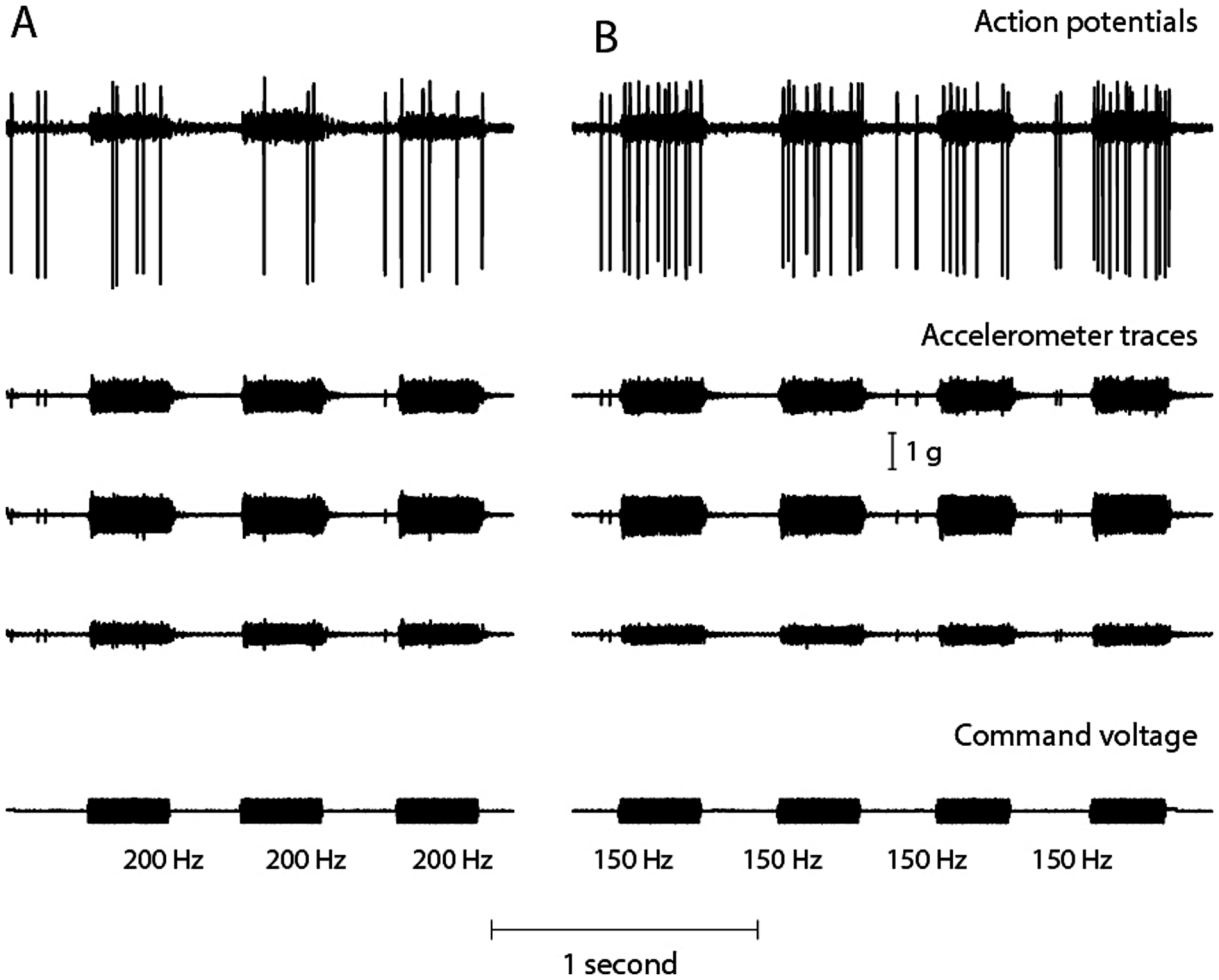
To show that low frequency skull vibration (100 Hz) activates canal neurons in animals with intact bony labyrinths. Time series of an anterior semicircular canal neuron with irregular resting discharge to low frequency bone conducted vibration at 200 Hz **(A)** and 150 Hz **(B)**. A triaxial linear accelerometer cemented to the animal’s skull was used to measure head acceleration (in three dimensions x, y, and z – shown beneath the neural activation) during stimulus application, which is shown by the command voltage in the bottom trace. At 150 Hz BCV, the increase in the neuron’s firing rate is closely locked to the onset and offset of the stimulus – the stimulus intensity corresponding to 0.35 g linear head acceleration. At the higher frequency of 200 Hz there is modest increase in firing rate although the stimulus magnitudes are identical. There was no activation at 500 Hz. Reproduced from (85) with permission of Springer Nature.

Recently it was possible to record artefact-free responses from single semicircular canal neurons to low frequency skull vibration (100 Hz BCV) in animals with intact normally encased labyrinths. These results show that canal afferent neurons (and also otolithic neurons) were activated by low frequency stimuli (67). There was no response to comparable vibration stimuli at 500 Hz (Figure 9). This low frequency activation in animals with normal encased bony labyrinths is the basis of another vestibular clinical test – skull vibration induced nystagmus (SVIN) where low frequency vibration of the mastoid induces a nystagmus in patients with unilateral vestibular loss (91). In SVIN testing, 100 Hz vibration is the optimum frequency, but after SCD high frequencies (up to 700 Hz) can generate SVIN (92) in accord with the canal neural data showing that canal neurons can be activated after SCD by high frequencies. In guinea pigs with an intact otic capsule, the canal neural response to frequencies less than about 200 Hz implies that low stimulus frequencies, below about 300 Hz, should not be used in cVEMP testing since they will activate canal neurons, and such activation will compromise the specificity of the cVEMP for indicating saccular function.

### Vestibular projections to SCM

Anatomical and physiological evidence shows that all five vestibular end organs have projections to the SCM and so all are potentially capable of producing an ipsilateral vestibular evoked myogenic potential on the SCM (19). However, the anatomical and physiological evidence of the presence of these projections says nothing at all about their function. The projections only become relevant when the particular end organ actually responds to a stimulus, i.e., when the afferent neuron increases its firing rate and so contributes to the response. Otherwise, the projection awaits such activation in order to have any role in generating the fast myogenic response. The important question is not the demonstration of a projection but the demonstration that the end organ actually responds to the stimulus. That is why the neural evidence is so crucial to the neural origin of the cVEMP and that is why simply listing anatomical and physiological evidence of the existence of ipsilateral input from other sense organs in the labyrinth (37) is not really relevant to the generation of the VEMP.

### ABR threshold as a reference for stimulus intensity

In retrospect, using the physical intensity of ACS or BCV at ABR threshold as a baseline reference level for stimulus intensity for vestibular research requires reconsideration. ABR threshold is the outcome of cochlear mechanical and neural operation, whereas in vestibular studies ABR threshold has been used to equilibrate physical stimulus intensities for vestibular (not cochlear) stimulation between animals and between species. The large species differences in cochlear function (as shown by extremely large differences in audibility curves and also ABR threshold values of rats vs. guinea pigs; Figure 4) bring into question the appropriateness of using a cochlear reference as the baseline for measuring and comparing the intensity of vestibular stimuli especially when that baseline is used to compare the results of stimulation between species. Sensitivity to sound in terms of audibility reflects cochlear function and varies between species. Using ABR threshold as a reference/baseline leads to extremely different physical sound pressure levels delivered to the vestibular neurons. The same number of dB above ABR threshold entails completely different physical intensities of vestibular stimulation in rats and guinea pigs.

### Experimental and clinical evidence that cVEMPs are saccular

Is the cVEMP a specific test of saccular function? The above has shown the physiological evidence from animal studies indicates that at standard frequencies and intensities used in clinical testing, any contribution from semicircular canals is negligible and experimental and clinical evidence agrees with that indication.

Tsubota et al. conducted experiments in monkeys where in one animal they sectioned the entire vestibular nerve and, in another animal, just the superior vestibular nerve (93). Both animals had normal cVEMPs to ACS before the surgical section. They measured the effect of this nerve sections on ACS evoked cVEMPs. Cutting the entire vestibular nerve abolished the cVEMP to clicks and also eliminated caloric nystagmus of the monkey, confirming that the cVEMP, like caloric nystagmus, is a vestibular response. If just the superior branch of the vestibular nerve was cut, the cVEMP to clicks remained, but the caloric response was eliminated, because afferents from the horizontal canal travel in the superior division of the VIII nerve and afferents from the saccular macula travel mainly in the inferior division (94). The preservation of cVEMPs after section of the superior division of the VIII nerve is strong evidence that the cVEMP is driven by the inferior vestibular nerve and in light of the above neural evidence, most likely from the saccular macula.

That result was complemented by results from 7 human patients during schwannoma surgery where the inferior vestibular nerve was electrically stimulated and recordings made of the cVEMP. Stimulation of the inferior vestibular nerve generated a cVEMP response in the ipsilateral SCM and no response in the contralateral SCM (95). These results of lesioning and electrical stimulation complement each other, and both show that the cVEMP response is saccular.

Sheykholeslami et al. reported cVEMP recordings from a human patient where there was total anatomical absence of all the semicircular canals bilaterally, whereas the remainder of the membranous labyrinth, including the otolithic structures, was intact (96). With no semicircular canals this patient showed clear cVEMPs to ACS stimulation, once again showing the selective saccular origin of cVEMPs and the negligible contribution of semicircular canal input to cVEMP under clinical testing conditions.

We now have the opportunity to identify whether activation of canal afferents which Zhu al reported in rat primary semicircular canal afferents (2011) has a significant role in clinical testing of otolith function. The opportunity arises because of the development of the video head impulse test (vHIT) of the function of all semicircular canals (97, 98). It is now well established using vHIT that some patients have all semicircular canals functioning normally but have reduced or absent utricular or saccular responses (oVEMPs or cVEMPs) (99–106). The fact that the canals are fully operational yet there is reduced or absent cVEMP points strongly to the fact that whatever canal neurons may be activated by sound or vibration, their contribution to standard cVEMP tests is negligible. Of course, the testing of cVEMPs requires careful attention to technical details in testing, such as SCM tension (2, 83), just as caloric testing requires careful attention to technical details. We acknowledge that multi frequency testing should be used to provide further conclusive evidence of absence of saccular function (107).

Kjaersgaard et al. claimed that cVEMPs in response to electrical stimulation of ampullary nerves in human patients supported the role of semicircular canal afferents in cVEMPs, whilst acknowledging the issue of current spread (37). The latter is more probable since current spread by electrical stimulation of labyrinthine sense organs (called co-stimulation) is so widespread that even electrical stimulation by cochlear implant electrodes generates oVEMPs and cVEMPs (108–110).

As we have stressed the cVEMP is a short latency response to a single stimulus rather than a change in firing during a maintained stimulus. It is the earliest part of the stimulus within the first few milliseconds which is vital to the generation of the response (39, 40), and it is at this onset there is an increased firing rate in irregular otolithic neurons. Recent evidence is revealing the mechanism by which the extremely fast synaptic transmission between type I receptors and irregular afferents occurs (35, 36). That fast transmission occurs due to resistive coupling – a form of membrane potential dependent electrical transmission – between the type I receptor and the calyx, reminiscent of the foresightful comment of Cazals et al. (31) noted above.

## Conclusion

Physiology and human clinical results show VEMP tests are specific tests of otolith function for 500 Hz and clicks at 100 dB nHL in the intact labyrinth. At this intensity in standard clinical testing there is likely little contribution if any, from semicircular canals. The evidence of the saccular activation by sound and vibration at clinical test frequencies and intensities and the failure of canal afferents to affect cVEMPs with intact bony labyrinths leads to the conclusion that clinical cVEMPs are a specific test of saccular otolith function. This is supported by strong evidence from stimulation and lesion studies for the primary role of the saccule in generating the cVEMP response to ACS.

We have shown that the apparent disagreement between guinea pig and rat data about canal activation by ACS stimuli is most likely due to the very high physical intensities used for the rat compared to the guinea pig. Comparable stimulus intensities for both rat and guinea pig show comparable results with minimal canal contributions at standard clinical testing levels.

A recent review stated, “All in all, common application and interpretation of the cVEMP as a specific test of saccular and inferior vestibular nerve integrity in clinical practice needs to be reconsidered, until more evidence is provided.” (37) p. 8. This statement is especially surprising since, as we have shown, that review made no mention of the wealth of available evidence showing saccular activation by sound. So, we reject that statement, because as we have shown there is a wealth of evidence showing that ACS cVEMPs specifically test saccular function. We have presented highly relevant physiological evidence, already published, on the saccular origin of cVEMPs, much of which was omitted from their review which was purportedly on the neural origin of VEMPs. The present study has shown what was missing from their review: the strong, unambiguous evidence for the saccular origin of cVEMPs and the negligible contribution from canal afferents to cVEMPs in standard clinical test situations.

### New and noteworthy

Does semicircular canal activation contribute to vestibular tests of otolith function (VEMPs) using sound or vibration with clinical testing conditions? The answer is no. The physiological evidence is that there is negligible contribution from semicircular canal activation when VEMP tests are conducted with standard clinical stimulus frequencies and levels. The one study (in rats) that reported extensive canal activation used very high stimulus intensities. When intensity is restricted to values used in the clinic, canal activation is minimal and VEMPs are specific tests of otolith function. In contrast to guinea pigs, rats have very high auditory thresholds for frequencies of sounds used for human clinical VEMP testing (500-1000 Hz) which brings into question the applicability of results of sound evoked vestibular responses from the rat for understanding human vestibular function.

## In memoriam

We dedicate this paper to the memory of Professor Toshihisa Murofushi. Toshi made many contributions to the understanding of vestibular function and was a leader in the field in Japan. In particular with regard to cVEMPs both in terms of basic physiology of vestibular afferents and clinical testing. He was a leader and a pioneer and Toshi was an exceptionally nice guy and everyone in the Sydney labs loved him, and we are sad at his loss. He persevered in the face of continued lack of results until things did work. He had undaunted, steadfast, focused attention to the question at hand. That is borne out by his papers reporting the guinea pig data and all the papers that he published since because when he went back to Japan, he became one of the world’s foremost experts on VEMPs. He had very firm ideas about their cause, and he was steadfast in defending his ideas. He really was a pioneer of vestibular physiology in Japan uniting clinical understanding with basic vestibular physiology. Toshi passed away on 13 May 2024. Thank you and Vale Toshi.
